# Acceptability of the RecoverEsupport Digital Health Intervention Among Patients Undergoing Breast Cancer Surgery: Qualitative Study

**DOI:** 10.2196/77567

**Published:** 2025-10-17

**Authors:** Emma Sansalone, Jennifer White, Alison Zucca, Owen James Morris, Mitch J Duncan, Stephen Smith, Priscilla Viana da Silva, Anna Palazzi-Parsons, Rebecca Wyse

**Affiliations:** 1 School of Medicine and Public Health, College of Health Medicine and Wellbeing, University of Newcastle Australia, University Drive, Callaghan, NSW, 2308, Australia, +61 405422098; 2 Population Health and Surgical and Perioperative Care, Hunter Medical Research Institute, New Lambton Heights, NSW, Australia; 3 Health Protection, Hunter New England Local Health District, Wallsend, NSW, Australia; 4 Calvary Mater Newcastle Hospital, Hunter New England Local Health District, Waratah, NSW, Australia; 5 School of Medicine and Public Health, College of Health, Medicine, and Wellbeing, University of Newcastle, Callaghan, NSW, Australia; 6 Centre for Active Living and Learning, University of Newcastle, Callaghan, NSW, Australia

**Keywords:** breast surgery, oncology, breast cancer, digital technology, patient-centred care, behavior, eHealth

## Abstract

**Background:**

Enhanced recovery after surgery guidelines aim to optimize perioperative care and improve recovery outcomes. The guidelines contain clinician- and patient-led recommendations for pre- and postoperative care, with patient-led recommendations, including smoking cessation, early mobilization, and early resumption of eating and drinking. While adherence to these recommendations can improve recovery outcomes, it is typically low, and many patients require support. Digital health interventions (DHIs) are increasingly accepted as useful tools in delivering individualized health care and have the potential to support adherence to enhanced recovery after surgery guidelines. Evidence suggests that intervention use is optimized when DHIs are considered acceptable to end users. RecoverEsupport is a DHI designed to support patient adherence to surgical recovery guidelines, following breast cancer surgery, intended as part of a blended approach with standard care.

**Objective:**

The study aimed to explore the surgical experiences and perceived acceptability of the RecoverEsupport DHI among former patients undergoing breast cancer surgery.

**Methods:**

This qualitative study, underpinned by a constructivist paradigm, explored the recovery experiences and acceptability of RecoverEsupport among women who had undergone mastectomy for breast cancer at a tertiary hospital in New South Wales (NSW), Australia. In total, 57 eligible patients were identified from medical records and invited to participate. Among them, 15 consented and were given access to the RecoverEsupport DHI for approximately 2 weeks. Around 11 participants then participated in a semistructured interview exploring their recovery experiences and feedback on the DHI. Interviews were transcribed and double coded prior to analysis using an inductive thematic approach.

**Results:**

Participants reported varied experiences of breast cancer surgery and expressed a consistent need for support, with many reporting uncertainty, anxiety, and limited information and physiotherapy support. RecoverEsupport was perceived as acceptable, with strong potential to reduce anxiety, address gaps in care, and provide reassurance. Participants valued the practical content, particularly physiotherapy exercise videos, which reinforced clinician advice and promoted confidence, autonomy, and self-efficacy. The intervention was seen as empowering patients to manage recovery and support their physical and emotional needs. All participants reported that they would recommend RecoverEsupport to others undergoing breast cancer surgery, highlighting its potential as a valuable adjunct to usual care.

**Conclusions:**

RecoverEsupport was perceived as a valuable adjunct to standard perioperative care. Four key themes were identified. Participants reported that the program addressed key gaps in information, physiotherapy access, and emotional support, while complementing clinical care. The intervention was seen to empower patients by enhancing knowledge, confidence, and self-efficacy, enabling a more active role in their recovery. It also provided reassurance during a vulnerable period. These findings highlight the potential of DHIs to support patients within constrained health care systems and enhance recovery outcomes.

## Introduction

Enhanced Recovery After Surgery (ERAS) guidelines are implemented by clinicians internationally to standardize and enhance perioperative patient care [1]. ERAS guidelines were initially developed for colorectal surgery [2] but now exist for over 30 different types of surgery [2], including for breast surgery following a cancer diagnosis [3]. ERAS guidelines consist of evidence-based recommendations for implementation by the treating clinicians and recommendations for behaviors, which patients should undertake themselves. ERAS patient-managed recommendations typically target preoperative behaviors (smoking and alcohol cessation, being physically active, and maintaining a healthy weight) and postoperative behaviors (early mobilization, early resumption of oral feeding and drinking, physiotherapy exercises, and opioid minimization) [4].

When implemented, the patient-managed ERAS recommendations are effective. For example, elective patients undergoing colorectal surgery who modified their behaviors had improved postoperative outcomes [5,6]. Ceresoli et al [7] demonstrated that postoperative complications were higher (~70%) among patients who did not adhere to the recommended early feeding, drinking, and mobilization behaviors, compared with patients who did adhere to the ERAS recommendations (~16%).

However, increasing demands on health care systems mean there is less capacity to support patients in implementing these patient-managed recommendations [8]. A recent systematic review of prehabilitation interventions for adult patients with cancer demonstrated that providing support directly to patients increases the likelihood of them engaging in perioperative behaviors [9]; however, this is not routinely practiced [10]. Most patients do not receive sufficient information and support about the patient-led ERAS recommendations in order to successfully perform these behaviors.

Current interventions fall short in educating and supporting patients about the patient-managed ERAS recommendations, highlighting the necessity for enhanced support systems. A systematic review (conducted in 2023) that explored patients’ experiences of ERAS found that many patients received generic educational materials that did not address their individual needs. In addition, limited opportunities for interaction with clinicians resulted in insufficient information and guidance about patient-managed ERAS recommendations. Emotional aspects of surgery recovery were often overlooked, and patients reported a lack of adequate follow-up care and support for managing recovery at home, a factor that slowed their recovery process.

These findings indicate the need for more patient-centered approaches in implementing ERAS programs to help improve patient outcomes and satisfaction. Patient-centered care is broadly defined as care that respects and responds to individual patient preferences, needs, and values and ensures that patient values guide all clinical decisions [11]. A recent umbrella review identified key elements of patient-centered care, including patient empowerment, recognition of patient individuality, and a biopsychosocial approach to care [11]. These elements align with core ERAS principles, which emphasize collaborative care and active patient participation.

In the surgical setting, these approaches play a critical role in improving patients’ understanding of their procedures, setting realistic expectations, and promoting behaviors, such as adherence to preoperative instructions, early mobilization, and self-management strategies that contribute to optimal recovery outcomes.

Digital health interventions (DHIs) can offer convenient, person-centered, and individualized health care that supports patient engagement, empowerment, improved self-management, and adherence to ERAS guidelines. They are also capable of delivering behavior change techniques directly to patients at a time and place of their choosing [12], empowering patients to actively self-manage their surgical recovery [13]. In addition, DHIs are cost-effective, widely accessible within the community, and can be centrally implemented, making them easy to update as needed [12].

As of 2024, more than 1.3 billion people worldwide used some form of digital health technology, including mobile health apps, wearable devices, telemedicine, and remote patient monitoring [14]. DHIs have the potential to address several pressing challenges faced by health care systems, such as improving information management, enhancing the quality of care, supporting continuity of services, and managing costs [12].

However, in order for patients to benefit from DHIs, it is essential that they use and engage with the intervention. Increasingly, studies have shown that higher engagement with a DHI leads to a larger magnitude of behavior change [15]. A systematic review (conducted in 2024) investigating factors associated with DHI engagement and adherence in patients with cancer showed that compared with low-frequency usage, high-frequency usage of DHIs was associated with better health outcomes, including an increase in treatment adherence, better quality of life, and improved symptom management [16]. In contrast, low engagement can limit the effectiveness of DHIs.

Previously documented barriers to DHI engagement include difficulty navigating the technology, lack of embedded motivational strategies included within the intervention, and insufficient personalization of content [17]. End-user acceptability testing is crucial to ensure that DHI solutions are effective, accessible, and aligned with the needs and preferences of end users [18].

Assessment of acceptability can include participants’ perceptions of the relevance, usefulness, and value of the DHI, particularly with respect to their own experience [19]. In this study, acceptability was defined as participants’ perceptions of the relevance, usefulness, and value of the RecoverEsupport intervention in relation to their surgical experience. Specifically, the evaluation focused on (1) whether participants felt the intervention aligned with their needs and experiences, (2) whether they believed it would have been beneficial during their own preparation and recovery, and (3) whether they would recommend it to others undergoing breast cancer surgery.

RecoverEsupport is a web-based program to support patients to enhance their surgical recovery by adhering to the patient-managed ERAS recommendations. The intervention is fully described in the published RCT protocol paper [17] and is briefly described below. The intervention incorporates evidence-based behavior change techniques in the form of information provision, skills training, self-monitoring and feedback, and supportive care prompts. Information and support are delivered directly to patients via text-based information, clinician videos, quizzes, behavioral checklists, and SMS text messaging or emails and are intended to be delivered as part of a blended approach in combination with standard care. Standard care at the study site includes preoperative education provided by nursing staff, printed educational materials, and routine postoperative follow-up.

RecoverEsupport was designed to complement this existing care pathway by providing additional, accessible, and patient-centered support throughout the perioperative period. The intervention is accessible to patients throughout the perioperative period and encourages patients to engage in 5 target behaviors, including early mobilization, early resumption of eating and drinking, minimizing opioid use, performing physiotherapy exercises, and practicing emotional self-care [20]. It is important to understand how patients can engage with and interact with RecoverEsupport to ensure the content, format, and approach meet the needs of the target audience—patients undergoing breast cancer surgery.

As part of the co-design development process for RecoverEsupport, researchers and clinicians (a surgeon, anesthetist, exercise physiologist, nurse unit manager, and specialist breast cancer nurses) collaborated in the development and refinement of intervention content. Two consumers from “Cancer Voices“—the peak body for cancer consumers within New South Wales (NSW), Australia [21]—were also involved.

The aim of this study was to explore the surgical experiences of women who had recently undergone breast cancer surgery and to evaluate the acceptability of a beta version of the RecoverEsupport intervention. Specifically, we sought to gain an understanding of patient experiences of breast cancer surgery preparation and recovery and how using RecoverEsupport would have influenced those things.

## Methods

### Study Design

This qualitative study is underpinned by a constructivist paradigm and adopts an inductive, reflexive thematic approach [22,23] grounded in interpretive analysis and emphasizes the active role of the researcher in theme development. This approach focused on the meaning and intentions people give to their own actions and their interactions with others [24,25]. This study used semistructured interviews and was informed by the COREQ (Consolidated Criteria for Reporting Qualitative Research) checklist [26].

### Study Setting and Recruitment

Participants were recruited using a purposive sampling approach to ensure the inclusion of individuals who had recently undergone breast cancer surgery and could therefore provide in-depth, relevant insights into their experiences with surgical preparation and recovery. Participants were eligible for inclusion if they (1) were female; (2) had previously undergone a surgical procedure involving a mastectomy for breast cancer between May 2022 and April 2023 at the Calvary Mater Hospital, Newcastle (a tertiary referral hospital in a regional metropolitan area of NSW, Australia [27]); (3) were English-speaking, (4) were living independently within the community (ie, were not living in a nursing home); and (5) had access to the internet and an email address. Participants were identified through searching medical records of the surgical department. The procedure descriptions (search terms) used to identify eligible participants in the medical record audit included “augmentation mammoplasty following mastectomy unilateral,” “Simple mastectomy bilateral,” “simple mastectomy uni,” “Subcutaneous mastecomt I bilateral,” and “Subcutaneous mastecomt I unilateral.” A total of 57 eligible former patients were identified from the medical records, and an invitation letter, participant information statement, and consent form were posted in November 2023. Participants could consent to the study via either QR code, web link, telephone, or post. Consenting participants were contacted by a research assistant to arrange a suitable time for a 30-minute telephone interview. Approximately 2 weeks prior to their scheduled interview, participants were emailed a web link to allow them access to the RecoverEsupport web-based program. They also received the accompanying correspondence, including a letter from a surgeon and prompts and reminders, that would typically be delivered as part of the intervention in addition to standard care. Participants were instructed to log into the RecoverEsupport web-based program and trial the intervention, consisting of 6 modules.

### Data Collection

Semistructured interviews (n=11), ranging from 30 to 45 minutes, were conducted by a female clinician-researcher (ES) and recorded. Interviews were guided by an interview schedule (Multimedia Appendix 1), and field notes were taken to capture contextual details and immediate reflections. The interview guide was developed by the researchers with input from 2 patient representatives from “Cancer Voices” [21]. Interviews began by asking participants to share their experiences of preparation for and recovery from surgery. Subsequent questions probed their experiences, perspectives, reactions, and feedback regarding the RecoverEsupport web-based program. To specifically assess acceptability, the interview guide targeted key elements, such as perceived relevance, usability, perceived benefits, and willingness to recommend the program to others. These elements were informed by established acceptability frameworks [28,29] and directly shaped the interview questions (refer to Multimedia Appendix 1). Preliminary themes were developed through iterative and reflexive engagement with the data, and data collection continued until the research team judged that sufficient depth and complexity had been achieved to address the research questions. This was guided by Braun and Clarke’s [30] meaning of saturation in reflexive thematic analysis, which refers to the point at which data collection provides sufficient depth, complexity, and insight to meaningfully address the research questions, rather than the absence of new codes or themes.

### Analysis

Interviews were transcribed verbatim and checked for accuracy by the primary researcher (ES). The inductive thematic approach involved (1) identifying units of meaning by reading the transcripts line-by-line, (2) grouping units into categories to assist with data retrieval, irrespective of the research question, and (3) examining relationships between codes to form themes. To guide the analysis and interpretation of participant perspectives, we used Social Cognitive Theory as an interpretive framework, with a particular focus on Bandura’s [28] theory of self-efficacy. This theory posits that individuals are more likely to engage in health-promoting behaviors when they believe they have the capability to perform them successfully. Incorporating this theoretical lens helped us to understand how participants’ confidence in managing their recovery may have influenced their engagement with the RecoverEsupport intervention. Two authors (ES and RW), one an accredited exercise physiologist with extensive clinical experience working in oncology and digital health, and the other a behavioral researcher specializing in digital health interventions, enacted this methodological process using NVivo 14 (Lumivero) qualitative data analysis software [31]. The lead researcher (ES) kept a reflexive journal documenting participant interactions and field notes, and the team engaged in ongoing peer debriefing to reflect on the research process and their positionality. ES and RW met regularly to consider how their professional vantage points influenced what was noticed or named in coding and to surface and mitigate potential biases. Following discussion with the broader team, initial codes were used to identify key categories, which were later grouped into themes. Coders captured exemplary quotes supporting each theme.

### Ethical Considerations

Approval for this project was obtained from the Human Research Ethics Committees of the Hunter New England Local Health District (2022/ETH02010), the University of Newcastle (H-2023‐0298), and the Calvary Mater Newcastle, Australia (2022/STE03757). All participants provided informed consent prior to participation. Participants’ privacy and confidentiality were protected, with all data deidentified during transcription and analysis. No compensation was provided for participation. No images or identifiable information of participants are included in the paper or supplementary materials. The study involved primary qualitative data collection, and all procedures adhered to ethical guidelines for participant autonomy, safety, and confidentiality.

## Results

### Overview

**Figure 1.**
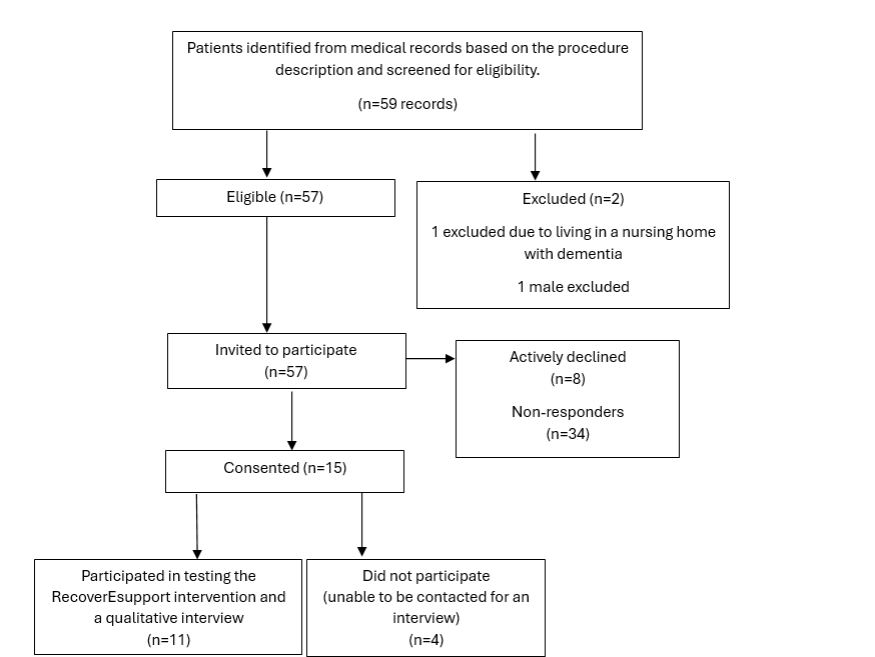
Participant recruitment flowchart for a qualitative study testing the RecoverEsupport digital intervention among patients who had recently undergone breast cancer surgery.

A total of 11 participants (Figure 1) were included in this study. Patients were identified from medical records based on procedure descriptions at a tertiary hospital in NSW, Australia (N=59). Two patients were deemed ineligible (one lived in a nursing home with dementia, one male patient). Of the 57 eligible patients invited to participate, 8 actively declined (one due to limited technological proficiency affecting internet access). Fifteen patients consented to participate, with 11 completing the intervention testing and a qualitative interview, and four unable to be contacted for an interview. Participant demographics and treatment characteristics are outlined in Table 1. Participants had an average age of 55 (SD 9.1) years. Among them, 6 (55%) were in a relationship, 10 (91%) were born in Australia, 6 (55%) were employed, and 2 (18%) held private health insurance. Overall, 5 (46%) participants underwent a single mastectomy, which was the most common surgical procedure performed. The number of surgeries throughout treatment varied, with 4 (36%) participants having had previous breast cancer surgeries.

**Table 1.** Participant demographics and treatment characteristics in a qualitative study of RecoverEsupport following breast cancer surgery (Australia, N=11, 2022–2023).

Characteristic	Participants
Age (years), mean (SD; range)	55 (9.1; 45-73)
Age at time of interview (years), n (%)	
40-49	3 (27.3)
50-59	5 (45.5)
more than 60	3 (27.3)
Marital status, n (%)	
Married	3 (27.3)
De facto	3 (27.3)
Divorced or separated	2 (18.2)
Single or widowed	3 (27.3)
Education level, n (%)	
Year 10 or below	3 (27.3)
TAFE (Technical and Further Education) or Vocational Training	2 (18.2)
Diploma or advanced diploma	4 (36.4)
Bachelor’s degree	2 (18.2)
Country of birth, n (%)	
Australia	10 (90.9)
Other	1 (9.1)
Employment status, n (%)	
Full-time	2 (18.2)
Part-time or casual	2 (18.2)
Retired	2 (18.2)
Self-employed	2 (18.2)
Unemployed	3 (27.3)
Private health insurance, n (%)	
Yes	2 (18.2)
No	9 (81.8)
Overall health (self-reported), n (%)	
Excellent	0
Very good	4 (36.4)
Good	4 (36.4)
Fair	1 (9.1)
Poor	2 (18.2)
Time since surgery (months), n (%)	
6-12	3 (27.3)
13-24	8 (72.7)
Surgery classification, n (%)	
Double mastectomy	2 (18.2)
Single mastectomy	5 (45.5)
Double mastectomy+immediate reconstruction	2 (18.2)
Single mastectomy+immediate reconstruction	2 (18.2)
Previous surgery^a^, n (%)	
Yes	4 (36.4)
No	7 (63.6)
Length of hospital stay (days), n (%)	
0-2	7 (63.6)
3-4	0 (0)
more than 5	4 (36.4)

a Refers to any previous surgical procedure, not limited to breast cancer–related surgeries.

**Figure 2.**
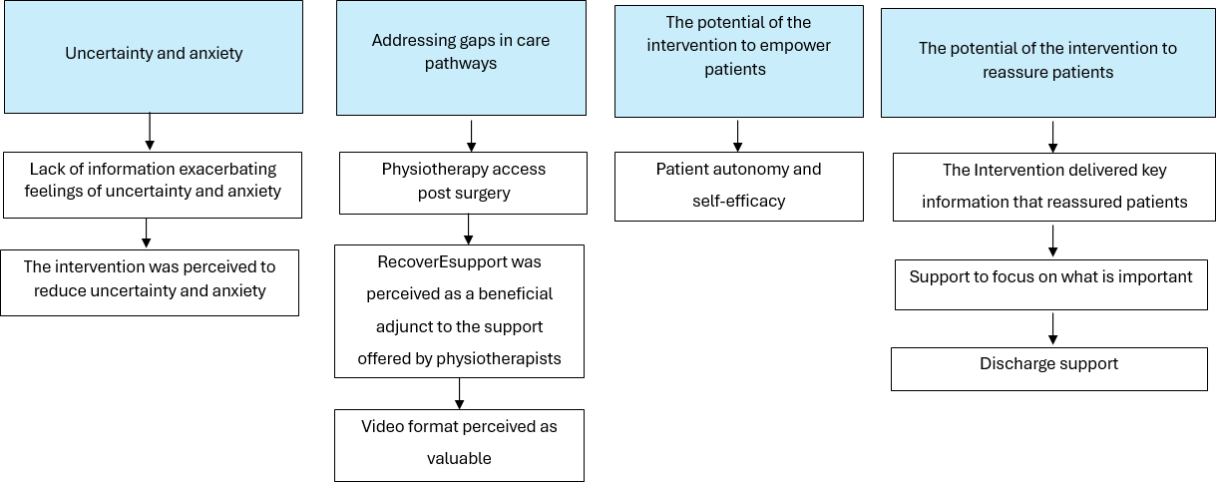
Overview of themes and categories describing patients’ perioperative experiences and perspectives on the acceptability of the RecoverEsupport digital health intervention.

The following themes were developed through iterative, reflexive analysis of the data and are outlined in Figure 2:

1. Theme 1. Uncertainty and anxiety
2. Theme 2: Addressing gaps in care pathways
3. Theme 3: The potential of the intervention to empower patients
4. Theme 4: The potential of the intervention to reassure patients

Themes were derived from qualitative interviews with women who had undergone breast cancer surgery between May 2022 and April 2023 at a tertiary hospital in NSW, Australia. Categories within each theme illustrate how participants perceived the intervention as reducing anxiety, complementing physiotherapy support, delivering valuable information in video format, and assisting with prioritization during recovery.

### Patients’ Perioperative Experience

All patients spoke at length about their experience with surgery and the care they received. These reflections provided context for their feedback about the RecoverEsupport intervention. When considering broader questions about intervention acceptability, participants often related their responses to their previous surgical experience. The number of previous surgeries varied between participants, with some undergoing multiple surgeries as part of their treatment for initial and recurrent breast cancer. Participants’ reports suggested that their initial experience of surgery shaped their approach and emotional response to future surgeries. In particular, many expressed feelings of heightened anxiety when their cancer returned, and that anxiety extended to the additional surgery that they required. Length of hospital stay also varied widely, with some participants being discharged within 18 hours while others remained in hospital for longer periods due to complications or the need for ongoing monitoring.

### Theme 1. Uncertainty and Anxiety

Many patients expressed feelings of uncertainty and anxiety during their treatment and recovery. This was typically compounded by the short time between their diagnosis and decision to undergo surgery. Feelings were further exacerbated when other life stressors were present, such as being a single mother and a carer to aging parents.

*I was very scared. I’m not married anymore. So I’ve got my kids at home with me. And I’m also my father’s carer. So it was a little scary to be going in for such a major surgery*.

Many participants reported situations during their inpatient stay where they felt uninformed about the surgical procedure and recovery process. A few participants reported struggling with knowing what to expect during and after their surgery. To address this, some participants proactively sought information from other sources, with mixed results. Feelings of uncertainty regarding what was going to happen and what they needed to be doing to manage their recovery from surgery often contributed to heightened anxiety.

*If it’s the unknown, we get nervous, we get anxious. And that’s what happened*.

The RecoverEsupport intervention was perceived to be a way of reducing anxiety during the perioperative period. All participants believed that the RecoverEsupport intervention would help alleviate feelings of anxiety. Providing information about the procedure was considered important and a useful way of managing patients’ expectations and reducing anxiety. However, it was acknowledged that due to the inherent nature of the experience, it would be unlikely to eliminate all anxiety.

*Anyone who’s diagnosed for the first time and is feeling very afraid and fragile, I think it [the RecoverEsupport program] would be good,*

*Interviewer: Would having access to this program have helped with your anxiety?*

*Participant: Definitely. Just the way it was all laid out. This was my first surgery, so even things like the surgical drain, I didn’t know what one of those was. I didn’t know that was going to be something that would be attached to me. So even just that little bit of a heads up. ‘Hey, you’re going to need to look after this’, and just the little things like that. So that you know a bit more*.

### Theme 2: Addressing Gaps in Care Pathways

Participants’ reports suggested that there was wide variability in access to physiotherapy, with some being unable to receive any postsurgery physiotherapy during their hospital stay. While a postsurgery exercise pamphlet was typically provided to patients, many were not sure how to address their specific recovery needs. For patients who didn’t receive any physiotherapy, some took the initiative to problem-solve and manage their recovery independently.

*Yes, [I got] a pamphlet … And it’s good, but it doesn’t really explain much. I had to figure it out, and I really needed a physio to come and tell me exactly how to do it*.

Overall, participants perceived physiotherapy staff to be busy, which contributed to feelings of anxiety about the support they needed for recovery. Notably, participants reported that physiotherapy staff were not available on the weekends. One participant reported feeling apprehensive about discharge in the absence of seeing a physiotherapist and reported they opportunistically sourced physiotherapy input.

*I saw a physio walking round the ward in the morning. I went home in the afternoon. And I said, “oh, are you coming to see me?” And she said, “You know you weren’t even on the list for me to be seen.” And so she gave me some exercises. But she didn’t have allocated time to spend with me*.

Many participants reported that they were unsure about postoperative mobility and rehabilitation exercises that they could be performing during their hospital stay. They expressed a desire for information and education on these exercises prior to surgery. Following the review of the RecoverEsupport intervention, participants reported its benefit as a countermeasure to the resource shortages that were identified through their perioperative experience. Participants viewed the RecoverEsupport intervention as a valuable addition, particularly in regard to postsurgery and post-discharge exercise. Participants also indicated that the intervention would increase the likelihood of them completing the target behaviors, such as performing the physiotherapy exercises.

*I think a bit of a visual cue [intervention strategy] is probably going to enforce or reinforce people to do their exercises*.

Participants especially appreciated the format in which the exercises were delivered (ie, demonstrated through a series of short videos). They considered this approach effective and beneficial, as this provided more information and detail than the existing resources.

*It was good actually seeing [the instructor] demonstrate the exercises as well. It told you how many times to do it every day. Don’t overdo it. They emphasise when they were showing, don’t push yourself too far, which some people might be inclined to do*.

### Theme 3: The Potential of the Intervention to Empower Patients

Participant overall reflections on their experience and their use of the RecoverEsupport intervention identified that they were keen to play an active role in their surgical recovery. However, most participants expressed that their perioperative experience lacked clear direction or information about what they could do to enhance their recovery. They particularly highlighted the physical and emotional strain of a breast cancer diagnosis and anticipation of surgery. Participants indicated that access to the RecoverEsupport intervention before their surgery had the potential to increase their knowledge about what to expect and what they could be doing to best prepare for surgery and where to access help if needed. Overall, participants found RecoverEsupport to be acceptable. They valued and appreciated the provision of reliable information, education, and support and thought this would enhance their knowledge and confidence in managing their own recovery, resulting in a sense of empowerment.  

*I feel as though it [RecoverEsupport] gives you a bit of confidence when you are lacking a lot of confidence. You’ve been put through the ringer physically and mentally even before your surgery with the diagnosis and things like that*.

Being able to compare their journey to what was expected or not reportedly allowed participants to know what to expect and identify areas where they might need to make changes.

*It pointed out what was normal and what was acceptable to expect after the operation. Also, it’s probably something I would’ve looked at the time to see, oh, okay, to remind me this is what I could be doing to help myself, this is what I should expect after the mastectomy with the drain as well*.

Participants reported that the RecoverEsupport intervention strengthened their autonomy as patients, empowering them to take a more active role in their recovery via education on self-care. They reported that this contributed to feelings of hopefulness throughout their recovery.

*I think it just gave people hope that you can get through it. That there are things you can do to help yourself, that it’s not all doom and gloom, that you can do things and come out the other end*.

Participants’ reports also suggest that the RecoverEsupport program promoted shared decision-making with clinicians by encouraging greater engagement in the recovery process. Through clear education on what to expect, participants indicated that they would have felt more equipped rather than relying solely on the next clinical encounter with a health care professional.

*It makes you feel a bit more part of the process rather than just doctors popping in and out, nurses popping in and out*.

For some participants, the RecoverEsupport intervention was perceived to strengthen their self-efficacy.

*You’ve seen what it does, how it helps. You’ve got the strength. Yes, just that little bit of power you’re taking back*.

### Theme 4: The Potential of the Intervention to Reassure Patients

Most participants felt that RecoverEsupport was beneficial in promoting feelings of reassurance. Access to clear, accurate information was perceived as central to managing expectations of the hospital admission by providing a clear sequence of each step and what to expect and reducing some of the uncertainty and fear.

*Rather than sitting in the waiting room shaking or panicking, you might just be like, “this is where we’re at. Next, they’re going to come out and grab me. Next, I go into the room and meet the anaesthetist and then next, I go to sleep. Then when I wake up, I'm in a recovery room.” So, I think that just helped … because for most people, hospital is a little bit scary, it’s not somewhere you’d go too often. So just knowing [helped], when I wake up, I'm going to be in a special room and then I'm going to be there a couple of hours, then I’ll be moved to the ward*.

During a time where there was potential to feel overwhelmed, participants reported that access to RecoverEsupport would have helped them better make sense of the large volume of information they had been given throughout their treatment and know where to focus their energy. For example, the video instructions were perceived to reinforce and complement clinician messaging, which participants acknowledged was important, particularly given the challenges of retaining and recalling information at this time (due to factors such as being in pain, distressed and anxious, and on medication). As such, the intervention was perceived as an opportunity to revisit and reinforce important information or overlooked topics.

*It [was] more of a reinforcement rather than … all this new information. And you trying to comprehend that whilst you're also maybe in a little bit of pain, on medication, things like that*.

Furthermore, participants felt that receiving detailed education would have motivated them to take more initiative, as it would have provided a clearer understanding of what was important for their recovery.

*I would have known what I should be doing. Like, they came and said, “oh you should be up.” I would have known personally that if I get up, the blood’s going to circulate better*.

Participants reported that RecoverEsupport provided essential information and support around discharge. The availability of this support in a web-based format was particularly helpful as it allowed patients to access and reinforce information at any time. This flexibility was especially beneficial for reviewing details that may have been forgotten during the stress of the presurgical consultations and during the hospital admission stay.

*I think just having that extra support at home once you get home. Because a lot of this stuff does go out of your head. But just having that then to reinforce everything when you get home*.

### Overall Participant Perspectives on Acceptability

All participants indicated that they thought the intervention would be acceptable to patients undergoing breast cancer surgery based on its relevance to their surgical experience, perceived usefulness in supporting preparation and recovery, and the value they attributed to it as a resource they would recommend to others. These indicators align with the study’s definition of acceptability, emphasizing the intervention’s relevance, usefulness, and overall value to patients.

*Look, if I had have had that in the beginning, it would have been such a lifesaver. I found it really good, and it covered everything that I would have [wanted] covered*.

Furthermore, when asked if they would recommend the intervention to others going through similar surgery, all participants agreed that they would. “*Most definitely.*”

One participant suggested that it would be beneficial for all types of surgery, not only breast surgery.

*I just think it’s something that would’ve helped me immensely, but I don't think it’s something that could just be restricted to breast cancer. I think there’s things in there that could be incorporated to all surgeries*.

## Discussion

### Principal Findings

The study aimed to explore the surgical experience and perceived acceptability of the RecoverEsupport DHI among women who had undergone breast cancer surgery. This study provides valuable insights into the perioperative experience of women undergoing breast cancer surgery and suggests that RecoverEsupport is not only acceptable to patients but also addresses key needs throughout the surgical journey. By exploring patient perspectives on the entire perioperative period, this study identified key features and functions of the intervention that were perceived to support patients in undertaking the patient-managed ERAS behaviors.

Participants perceived that the RecoverEsupport intervention would reduce the anxiety they experienced regarding their surgery. By offering real-time feedback and answers to common concerns, participants felt that the intervention would help alleviate anxiety and uncertainty. In times of uncertainty, people often engage in anticipatory coping strategies, such as creating worst-case scenarios in their minds to anticipate possible outcomes [32]. Access to clear and accurate information can help them prepare both mentally and physically, replacing anxiety with a sense of control and readiness. This is consistent with previous research demonstrating that preoperative interventions can reduce anxiety among people undergoing surgery [6]. Most participants perceived some shortcomings or gaps in their perioperative care, either before surgery, during their hospital admission, or after discharge. Consistent with previous research, gaps were largely attributed to a lack of resources within the health care system. Study participants identified that these gaps contributed to feelings of uncertainty and increased anxiety and that the addition of the RecoverEsupport program would lead to patients feeling more informed and confident and less anxious.

It is well-established that patients want to be actively involved in their recovery, make informed decisions, and understand the steps they need to take to enhance their recovery [33]. This study identified that RecoverEsupport is a resource well-suited to this end, with participants perceiving it to empower them and encourage self-management. RecoverEsupport also has the potential to increase patients’ confidence in their ability to manage their recovery by providing access to reliable information and support whenever it is needed. Participants expressed that using RecoverEsupport would make them more likely to engage in the recommended postoperative behaviors (ie, physiotherapy exercises and mobilization) and therefore optimize their recovery. This aligns with Bandura’s [28] theory of self-efficacy, which highlights that individuals are more likely to engage in health-promoting behaviors when they believe they can successfully perform them. These results were also consistent with several studies that found perioperative interventions for patients with breast cancer enhanced empowerment and self-efficacy, contributing to better postoperative outcomes [34-36].

RecoverEsupport has the potential to contribute to better recovery experiences by complementing the care provided by clinicians. Rather than replacing traditional clinical interactions, participants in this study felt that the RecoverEsupport DHI could effectively complement and reinforce the advice and guidance given by health care providers. RecoverEsupport was viewed as a valuable tool for providing ongoing support, reminders, and tailored information and education, which could help patients feel more supported throughout their surgical journey, particularly between medical appointments. Their responses highlight the potential for blended care processes (whereby the existing care and support from clinicians is supplemented through digital intervention and support) to more comprehensively address the full spectrum of patient needs.

Acceptability is a frequently used but poorly defined term. Sekhon et al [29] have developed a theoretical framework for understanding acceptability, which identifies 7 constructs. The themes identified in this study align with key constructs from this framework. For example, participants liked and felt positively toward the intervention and acknowledged they would have liked to have had it when they were going through their surgeries, aligning with the “affective attitude” construct (ie, “how an individual feels about the intervention” [29]). Furthermore, participants described how they felt empowered by the intervention and how it would have helped them prepare for and recover from their surgery, aligning with the construct of “perceived effectiveness” (“the extent to which the intervention is perceived as likely to achieve its purpose”). Intervention coherence is defined as “the extent to which the participant understands the intervention and how it works.” [29]. Feedback that the participants understood the links between the intervention components and the outcomes, that is, they linked watching the intervention videos to their own behavior and being more likely to complete their postsurgery exercises, suggests that the intervention also addressed this acceptability construct.

This is the first study to explore the acceptability of a DHI to enhance recovery from breast cancer surgery across the perioperative period, focusing on patient-managed ERAS behaviors. However, other DHIs have been developed to support patients with breast cancer through chemotherapy and radiation treatments [37], and previous qualitative research has identified that these types of DHIs were acceptable and were perceived to improve patient knowledge and quality of life and reduce levels of anxiety [37]. Specifically, a scoping review (conducted in 2024) highlighted that DHIs can support patient education and decision-making around treatment options [38]. Similarly, another study found that DHIs effectively improve quality of life and self-efficacy among breast cancer survivors [39]. However, there is a noticeable gap in DHIs specifically for patients undergoing breast cancer surgery across the perioperative period. While some interventions, such as web-based exercise programs, have been evaluated for their acceptability postsurgery, comprehensive DHIs specifically designed to support patients during the perioperative period are limited [40].

### Strength and Limitations

The strength of this study lies in the exploration of a heterogeneous sample of women from various sociodemographic backgrounds and a wide age range. To enhance the credibility and trustworthiness of the study, we used several strategies throughout the research process. Interviews were transcribed verbatim and checked for accuracy, and thematic development involved iterative, reflexive engagement with the data. We maintained a detailed audit trail and engaged in regular team discussions to support analytical rigor. We acknowledge the potential for reporting bias and that responding participants may have had different experiences than nonresponders, including access to other hospitals where differing models of care may exist. Variation in patients’ perioperative experiences may have influenced their reaction to the RecoverEsupport intervention. We also acknowledge the lack of representation from culturally and linguistically diverse carers and women residing in rural and remote settings. Future research should include a more diverse range of participants to explore how the experiences and needs of those going through cancer treatment may differ and how this digital health resource and others like it are perceived.

An additional limitation relates to the technological format of the RecoverEsupport intervention. While digital delivery offers advantages, such as convenience and scalability, it may also present accessibility barriers for certain patient groups. These include older adults, individuals with limited digital literacy or access, those with low health literacy, and people with neurodiverse conditions, such as dyslexia. Since health literacy was not assessed in this study, it remains unclear how acceptable and usable the intervention would be across diverse populations, including socioeconomically disadvantaged or digitally excluded patients. These factors highlight the need for further research to explore tailored strategies that ensure equitable access and engagement with digital health interventions.

### Conclusions

This study demonstrates that RecoverEsupport was perceived as acceptable and was perceived to address key patient needs throughout the surgical journey. It was seen as an acceptable addition to the standard care provided and was perceived to complement clinical care and bridge gaps in care that patients experienced. Importantly, it was perceived to assist in empowering patients to take a more active role in their recovery and to provide emotional reassurance during a vulnerable time. In the context of strained health care systems and the growing demand for patient-centered care, these findings highlight the potential for scalable digital interventions to complement clinical services, reduce care gaps, and enhance recovery experiences. As such, integrating DHIs into standard perioperative pathways may represent a critical step toward more responsive, personalized, and sustainable models of surgical care.

## Supplementary material

10.2196/77567 Multimedia Appendix 1 Acceptability interview guide.

## Abbreviations

COREQ: Consolidated Criteria for Reporting Qualitative Research
DHI: Digital health intervention
ERAS: Enhanced Recovery After Surgery
NSW: New South Wales

## References

[1] McLennan E, Oliphant R, Moug SJ (2019). Limited preoperative physical capacity continues to be associated with poor postoperative outcomes within a colorectal ERAS programme. Ann R Coll Surg Engl.

[2] Guidelines. ERAS Society.

[3] Breast. ERAS Society.

[4] Temple-Oberle C, Shea-Budgell MA, Tan M (2017). Consensus review of optimal perioperative care in breast reconstruction: enhanced recovery after surgery (ERAS) society recommendations. Plast Reconstr Surg.

[5] Gustafsson UO, Hausel J, Thorell A (2011). Adherence to the enhanced recovery after surgery protocol and outcomes after colorectal cancer surgery. Arch Surg.

[6] Greer NL, Gunnar WP, Dahm P (2018). Enhanced recovery protocols for adults undergoing colorectal surgery: a systematic review and meta-analysis. Dis Colon Rectum.

[7] Ceresoli M, Pedrazzani C, Pellegrino L (2024). Early non compliance to enhanced recovery pathway might be an alert for underlying complications following colon surgery. Eur J Surg Oncol.

[8] Poletti B, Stringer G, Furness K (2024). Patient experience pre-implementation of an enhanced recovery after surgery protocol: a qualitative investigation. J Multidiscip Healthc.

[9] Scriney A, Russell A, Loughney L, Gallagher P, Boran L (2022). The impact of prehabilitation interventions on affective and functional outcomes for young to midlife adult cancer patients: a systematic review. Psychooncology.

[10] Wang D, Hu Y, Liu K (2023). Issues in patients’ experiences of enhanced recovery after surgery (ERAS) : a systematic review of qualitative evidence. BMJ Open.

[11] Grover S, Fitzpatrick A, Azim FT (2022). Defining and implementing patient-centered care: an umbrella review. Patient Educ Couns.

[12] WHO guideline recommendations on digital interventions for health system strengthening. World Health Organization.

[13] van der Meij E, Anema JR, Otten RHJ, Huirne JAF, Schaafsma FG (2016). The effect of perioperative E-health intervention on the postoperative course: a systematic review of randomised and non-randomised controlled trials. PLOS ONE.

[14] Digital health users worldwide 2029. Statista.

[15] Eaton C, Vallejo N, McDonald X (2024). User engagement with mHealth interventions to promote treatment adherence and self-management in people with chronic health conditions: systematic review. J Med Internet Res.

[16] Montalescot L, Baussard L, Charbonnier E (2024). Factors associated with digital intervention engagement and adherence in patients with cancer: systematic review. J Med Internet Res.

[17] Borghouts J, Eikey E, Mark G (2021). Barriers to and facilitators of user engagement with digital mental health interventions: systematic review. J Med Internet Res.

[18] Williams CJ, Varnfield M, Stott A, Duff J (2024). Design overview and usability of the codesigned My Surgical Pathway E-health tool for supporting patient self-managed surgical preparation and recovery. Perioper Care Oper Room Manag.

[19] Monitoring and evaluating digital health interventions. World Health Organization.

[20] Sansalone E, Zucca A, Duncan MJ (2025). Study protocol of a pilot randomised controlled trial assessing the feasibility and acceptability of RecoverEsupport: a digital health intervention to enhance recovery in women undergoing surgery for breast cancer. BMJ Open.

[21] Cancer Voices Australia.

[22] Braun V, Clarke V (2022). Thematic Analysis: A Practical Guide.

[23] Braun V, Clarke V (2019). Reflecting on reflexive thematic analysis. Qual Res Sport Exerc Health.

[24] Pilarska J, Pabel A, Pryce J, Anderson A (2021). Research Paradigm Considerations for Emerging Scholars.

[25] Smith JK Interpretive inquiry: a practical and moral activity. Theory Pract.

[26] Tong A, Sainsbury P, Craig J (2007). Consolidated criteria for reporting qualitative research (COREQ): a 32-item checklist for interviews and focus groups. Int J Qual Health Care.

[27] Remoteness areas. Australian Bureau of Statistics.

[28] Bandura A (1978). Reflections on self-efficacy. Adv Behav Res Ther.

[29] Sekhon M, Cartwright M, Francis JJ (2017). Acceptability of healthcare interventions: an overview of reviews and development of a theoretical framework. BMC Health Serv Res.

[30] Braun V, Clarke V (2021). One size fits all? What counts as quality practice in (reflexive) thematic analysis?. Qual Res Psychol.

[31] About NVivo. NVivo.

[32] Aust H, Rüsch D, Schuster M, Sturm T, Brehm F, Nestoriuc Y (2016). Coping strategies in anxious surgical patients. BMC Health Serv Res.

[33] Powell R, Davies A, Rowlinson-Groves K, French DP, Moore J, Merchant Z (2023). Impact of a prehabilitation and recovery programme on emotional well-being in individuals undergoing cancer surgery: a multi-perspective qualitative study. BMC Cancer.

[34] Kim SH, Choe YH, Han AR (2020). Design of a randomized controlled trial of a partnership-based, needs-tailored self-management support intervention for post-treatment breast cancer survivors. BMC Cancer.

[35] Zhang Q, Wang L, Yang J, Liu W, Hong C (2025). Effects of an mHealth-based psychological resilience intervention on psychological resilience, negative emotions and self-efficacy in breast cancer patients during postoperative chemotherapy. Lancet Reg Health West Pac.

[36] Ye YY, Cao YC, Lu XJ, Xiang X (2025). Impact of Rosenthal effect-based nursing intervention on self-care ability and hope level in patients undergoing breast surgery. BMC Surg.

[37] Singleton AC, Raeside R, Hyun KK (2022). Electronic health interventions for patients with breast cancer: systematic review and meta-analyses. J Clin Oncol.

[38] Kirsch EP, Kunte SA, Wu KA (2024). Digital health platforms for breast cancer care: a scoping review. J Clin Med.

[39] Pimentel-Parra GA, Soto-Ruiz MN, San Martín-Rodríguez L, Escalada-Hernández P, García-Vivar C (2023). Effectiveness of digital health on the quality of life of long-term breast cancer survivors: a systematic review. Semin Oncol Nurs.

[40] Lee K, Kim S, Kim SH (2023). Digital health interventions for adult patients with cancer evaluated in randomized controlled trials: scoping review. J Med Internet Res.

