# Supplementary material for: Acceptability of the RecoverEsupport Digital Health Intervention Among Patients Undergoing Breast Cancer Surgery: Qualitative Study
Source: J Med Internet Res. 2025 Oct 17;27:e77567. doi: 10.2196/77567 (PMC12537582; doi:10.2196/77567)
Supplement: Multimedia Appendix 1 [file jmir-v27-e77567-s001.doc]

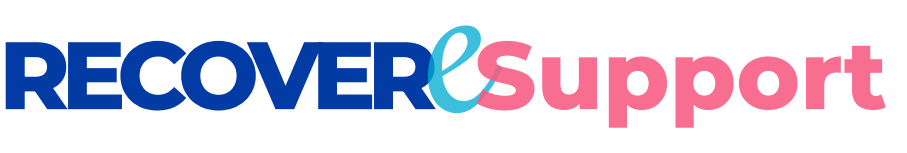


**Indicative telephone script:**

Good morning/afternoon. My name is _______. I'm calling from the University of Newcastle about the RecoverEsupport study. May I speak with _________ please?

**If person is NOT there**

Is there a better time I could call back and reach______________ (*persons first name*)?

LOG DETAILS

Thank you.

**If person IS there**

Hello ___(*first name).* I’m calling to arrange an interview with you to get your feedback about the RecoverEsupport program. The interview will take approximately 30 minutes. Is now a good time for the interview?

**If YES, now IS a good time for the interview.**

As part of the study, we will be recording the interview. Is this still ok?

**If NO, now is NOT a good time for the interview.**

That’s fine. When would be the best time for me to call you back? LOG DETAILS

**OR: NO, not interested in participating**

That's fine. I will record it on my end now. Thanks for your time. *END CALL*

**If yes, OK to audiotape the interview**

Great. Thanks. I will tell you when I am turning the tape on and off.

**Thank you for reviewing the ‘RecoverEsupport’ online program**

I’m going to refer to that program and ask your thoughts on it.

**Introduction**

- Before we begin, I need to remind you that your participation is voluntary, and you /have the option of terminating this interview at any time without giving me a reason. The interview should take about 30 minutes, but we do not have to finish it all in one go. About every 5-10 minutes I will check whether you’re happy to continue. There are no right or wrong answers in today’s interview – we are interested in your thoughts and opinions. Do you have any questions before we begin?

**I am now turning ON the tape recorder. To ensure your confidentiality, please try not to use names during the recording.**

START AUDIOTAPE

**QUAL:**

**General questions**

**Firstly, I’ll start by asking about the treatment you received.**

1. Could you tell me a bit about your experience with breast cancer surgery?

**Now, I’d like to ask you about your experience of trialling the ‘RecoverEsupport’ program.**

1. *Did you trial the RecoverEsupport program? (if yes) Can you tell me about your experience trialling it over the past few weeks?*
2. What were your overall impressions of the RecoverEsupport program?
3. Did you watch any of the videos? (if yes) What did you think about the videos?
4. Did you read the written information (if yes) What did you think about the written information in the program?
5. (Prompt: was it easy / difficult to understand?)

*If indicated some sections not easy to understand:* - Can you remember which sections were not easy to understand? Do you have any suggestions for how this information could be better presented?)

1. What did you think about the amount of information presented?

(prompt): Would you have preferred more or less detail for any sections?

(Prompt): Were there any things that you found more helpful than others?

(Prompt: Could you tell me more about that?)

1. Did you do the quiz questions? (If yes) What did you think of the quiz questions?
   (Prompt: too many, too simple, Did they help reinforce what you had just learned?)
2. Did you read the surgeon’s recommendation letter (if yes) What did you think about it?
3. Did you read the discharge letter to the GP (if yes) What did you think about it?
4. Did you look at the exercise log (if yes) What did you think about it?
5. Did you look at the physio exercises (if yes) What did you think about them?
6. Did you look at the daily diary/checklist (if yes) What did you think about it?
7. What did you think of the emails you received? (tone, length, amount)
8. Overall, how relevant was the online program to your experience of surgery?
9. *Did you have any questions after using the RecoverEsupport program? (Prompt: what were they?*
10. Is there any other information or advice that you thought was missing from the program that would be beneficial for patients to know in future?
11. How could we improve the program? For example, any other features that you think would help patients feel better prepared for surgery or to manage their recovery?
12. Overall, how useful do you think this program would have been to you, if you had had access to it when you were undergoing surgery? [prompt - before the breast surgery, have you experienced any other major surgeries that may have helped you better understand the surgical journey?]
13. Would you recommend the program to other people having surgery? Yes / No / Don’t Know (and why do you say that?)
14. What do you think were the key messages of the RecoverEsupport program?
15. Based on the RecoverEsupport program, what would you say were the most important things you could do:
16. to prepare for your surgery?
17. to help your recovery whilst in hospital ?
18. to help your recovery after discharge from hospital?
19. Can you recall the 5 recover E’s?

*QUANT:*

**Finally, could I ask some demographicquestions about you? This information will help to describe who has participated in this study.**

**ABOUT YOU**

**Finally, could I ask some personal questions about you and your cancer journey? This information will help to describe who has participated in this study. Are you OK to continue?**

| ***About you*** | | |
| --- | --- | --- |
| **A2.** | **What is your date of birth?** | ***___ ___ / ___ ___ / ___ ___ ___ ___***  Day Month Year |
| **A3.** | **What is your present marital status?** | * Single, never married  * Married/Living with partner  * Separated or divorced  * Widowed |
| **A4.** | **What is the highest level of education you have completed?** | * Year 10/School Certificate or lower  * Higher School Certificate  * Trade or vocational training (e.g. TAFE or college)  * Bachelor degree  * Postgraduate Degree  * Other |
| **A5.** | **What country were you born in?** | * Australia  * United Kingdom  * New Zealand  * Italy  * China  * India  * Other (please specify) ______________________________ |
| **A6.** | **What best describes your employment status at this time?** | * Full-time work  * Part-time or casual work  * Home duties  * Unemployed  * Retired  * Disability pension  * Other (please specify): ____________________________________ |
| **A7.** | **Are you of Aboriginal or Torres Strait Islander origin?** | * No  * Yes, Aboriginal  * Yes, Torres Strait Islander  * Yes, both Aboriginal and Torres Strait Islander |
| **A8.** | **Do you have private health insurance?** | * Yes  * No |
| **A9.** | **In general, how would you rate your overall health?** | * Excellent  * Very good  * Good  * Fair  * Poor |

**SECTION B: YOUR CANCER JOURNEY**

This section asks questions about your cancer and treatment you have received.

| ***Diagnosis and treatment*** | | | | | |
| --- | --- | --- | --- | --- | --- |
| **B1.** | **How long ago were you diagnosed with breast cancer?** | * Less than 1 month  * 1-2 months  * 2-3 months  * 3-4 months  * 4-5 months  * 5-6 months  * More than 6 months ago | |  | |
| **B4.** | **Approximately when did you have breast cancer surgery?** | | ***___ ___ / 20__ __***  Month Year | |  |
| **B5.** | **What type of surgery did you have?** | | * Mastectomy – no reconstruction  * Mastecomy and immediate reconstruction  * Mastecomy and then a delayed reconstruction  * Other (please specify) | |  |
|  | Prior to your surgery, did you access any information to help you prepare for your surgery?    ) | | • Cancer Council Website/Booklets  • Breast Cancer Network Australia Website  • McGrath Foundation  • Information provided from the Calvary Mater hospital  • Other (please specify | |  |
|  | **How many days were you in hospital?** | | Please specify | |  |

| **Did you do anything to prepare for your surgery? Please specify.** |  |
| --- | --- |

**OK. So that’s the end of the interview. Thank you for taking the time to help us and provide feedback on the web-based program. I am very grateful for your participation. We will use your feedback to further develop the program for future use.**

Is there anything else you think we should know?

Do you have any questions about the interview?

Would you like a copy of the transcript from this interview? If yes, what is your email?

If you have any questions or concerns about the study, please don’t hesitate to call me or Dr Rebecca Wyse on our free call number. Would you like our number?

IF YES.

Our number is (02) 4042 0964

**Once again, thank you very much for your help.**

**END INTERVIEW**
